# Supplementary material for: Molecular Mechanism of Food-Derived Polyphenols on PD-L1 Dimerization: A Molecular Dynamics Simulation Study
Source: Int J Mol Sci. 2021 Oct 10;22(20):10924. doi: 10.3390/ijms222010924 (PMC8535905; doi:10.3390/ijms222010924)
Supplement: Supplementary file 1 [file ijms-22-10924-s001.zip › SI.pdf]

Supplementary Materials

Molecular Mechanism of Food-Derived Polyphenols on PD-L1 Dimerization: A Molecular Dynamics Simulation Study

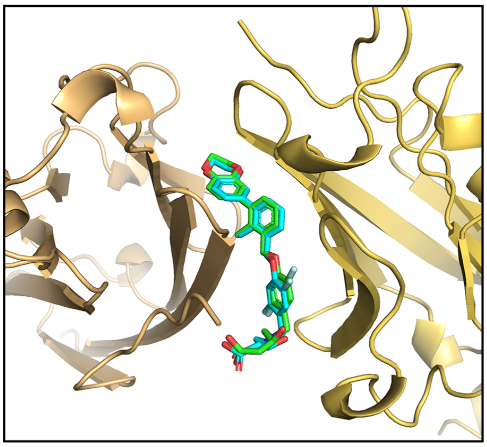

Figure S1. Overlap of the vina-docked pose and native conformation from the crystal structure (PDB ID: 5N2F). The docked pose and native confirmation are coloured cyan and green, respectively. $_{A}$PD-L1 and $_{B}$PD-L1 are coloured orange and yellow, respectively.

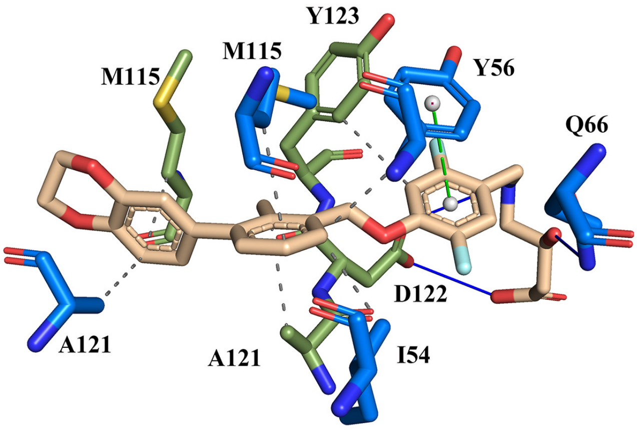

Figure S2. Binding modes and interactions between the docked BMS-200 and PD-L1 dimer. $_{A}$PD-L1 and $_{B}$PD-L1 are coloured green and blue, respectively. BMS-200 is coloured beige. H bond, hydrophobic, and Π-stacking interactions are also shown.
